# Status epilepticus following local anesthesia in a previously healthy adult

**DOI:** 10.1186/s13104-016-2100-9

**Published:** 2016-06-10

**Authors:** Rana Alnasser Alsukhni, Mohamed Sourat Ghoubari, M.Taher Farfouti, Yasmin Adib Aboras

**Affiliations:** Division of Neurology, Department of Internal Medicine, Aleppo University Hospital, Aleppo, Syria; Division of General Internal Medicine, Aleppo University Hospital, Aleppo, Syria; Head of Neurology Unit, Aleppo University Hospital, Aleppo, Syria

**Keywords:** Status epilepticus, Local anesthesia, Healthy adult

## Abstract

**Background:**

Local anesthesia could result in lethal complications if injected in highly vascularized area. Dentist should take care to avoid such complications.

**Case presentation:**

We present a case of 15 year old girl with a coma following convulsive status epilepticus which developed after inferior alveolar nerve blockade by a dentist. The patient was admitted to the intensive care unit ICU and recovered within several days.

**Conclusion:**

This case is reported to tell both of dentists and medical staff that although it is uncommon, such complications of local anesthesia should be in mind to be avoided and managed promptly if happened.

## Background

Systemic toxicity of local anesthesia can occur after administration of an excessive dose, with rapid absorption, or occur because of accidental intravascular injection [1, 2].

Of these, intravascular injection is probably now the most common cause of systemic toxicity and the only one that cannot always be prevented by proper dosage selection and administration technique [3].

Systemic toxicity of local anesthetics is typically manifested as central nervous system (CNS) toxicity (tinnitus, disorientation, and ultimately, seizures) or cardiovascular toxicity (hypotension, dysrhythmias, and cardiac arrest).

True overdosage is quite rare in dentistry, except in small children, because of the low doses employed. Nevertheless, case reports have documented unexpected convulsions and death in healthy adult patients receiving routine intraoral injections [4].

DeToledo reported a case of lidocaine-induced seizures in patients with history of epilepsy [5]. Brown DL1 reported several cases for seizures and cardiovascular changes resulting from local anesthetic in patients undergoing brachial plexus, epidural, and caudal regional anesthetics [6].

Menif reported three cases of lidocaine toxicity in infants secondary to local anesthesia administered in the community for elective circumcision [7].

This case, however, describes a status epilepticus in a previously healthy young female after a probable intra-arterial injection of lidocaine and adrenaline for local intraoral anesthesia.

## Case presentation

A 15 year old girl presented to the ER with coma following several tonic–clonic convulsions.

The patient was previously healthy with no personal or familial medical history. During her visit to a dentist for endodontic treatment, the dentist injected 1.5 ml of 2 % lidocaine (the total dose was 30 mg) and epinephrine 1:100,000 for local anesthesia. The solution was injected on the left side by an expert hand without prior aspiration.

The injection lasted about 2 min. By the end of the injection, the girl collapsed and several intermittent tonic–clonic convulsions ensued. These lasted about 30 min and were followed by deep coma.

### On examination

Glasgow coma scale (GCS) was three, brainstem reflexes were intact, and no fever was noticed. Vital signs were within normal limits.

Her left cheek and gingiva were edematous and a giant gingival hematoma was noticed.

Optic disc examination showed no edema.

Electrocardiogram (ECG) was normal except for sinus tachycardia (120 beat per minute).

Electroencephalography (EEG) showed diffuse slow activity, predominately theta rhythms, with no signs of continuous status epilepticus.

Brain computed tomography (CT) revealed reduced white–gray matter differentiation with effacement of cerebral sulci which was compatible with cerebral edema.

**Fig. 1:**
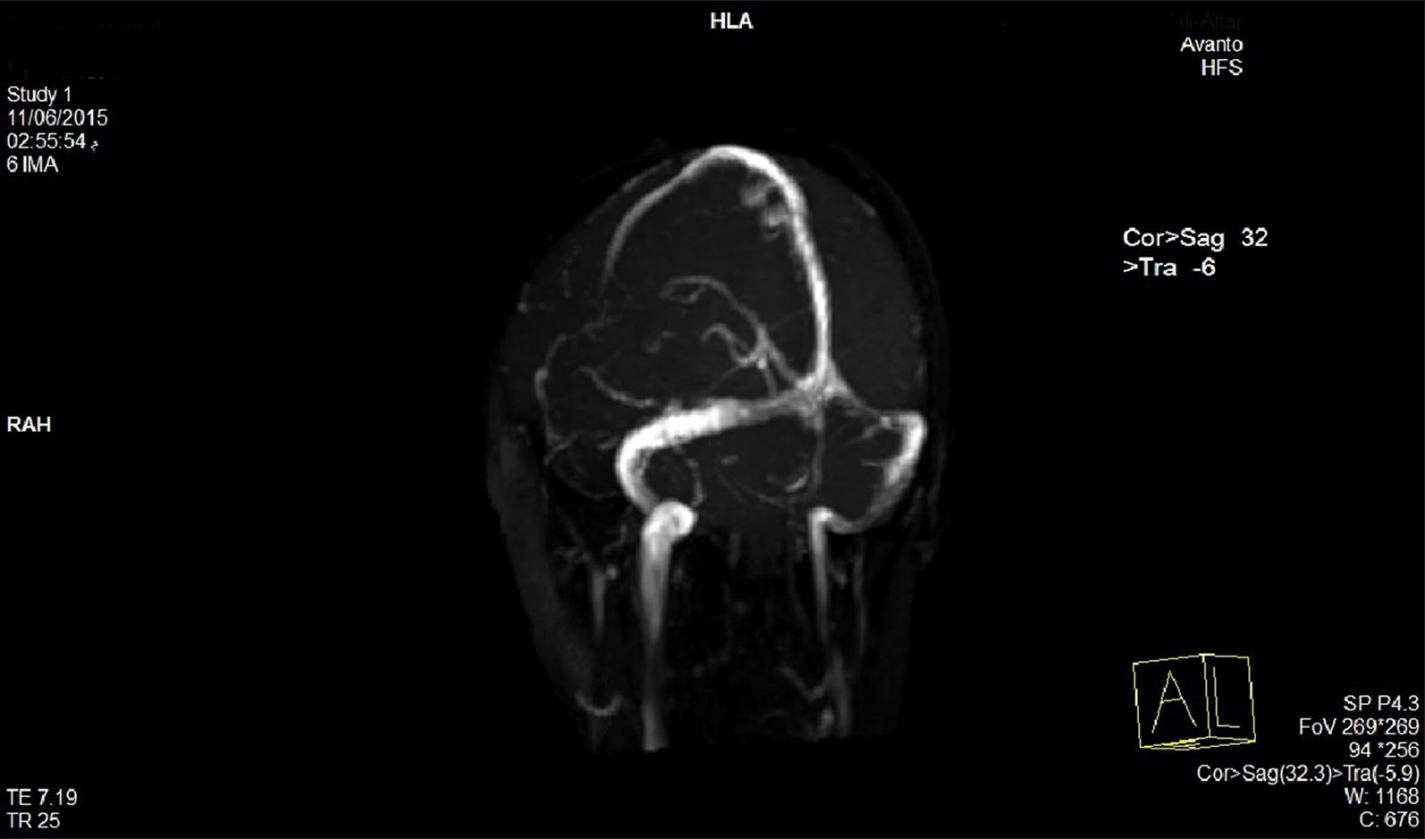
Magnetic resonance venogram demonstrates patent cerebral venous sinuses

**Fig. 2:**
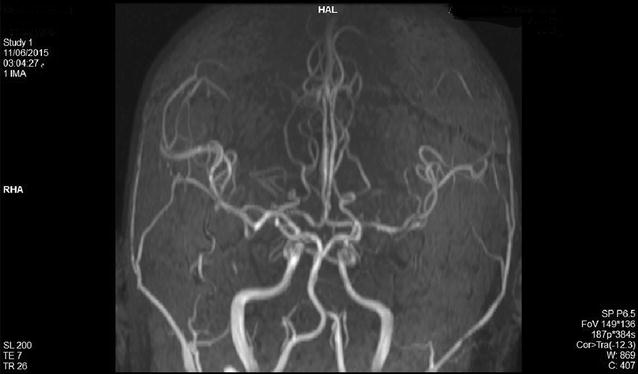
Magnetic resonance angiogram (MRA) shows normal cerebral arteries

**Fig. 3:**
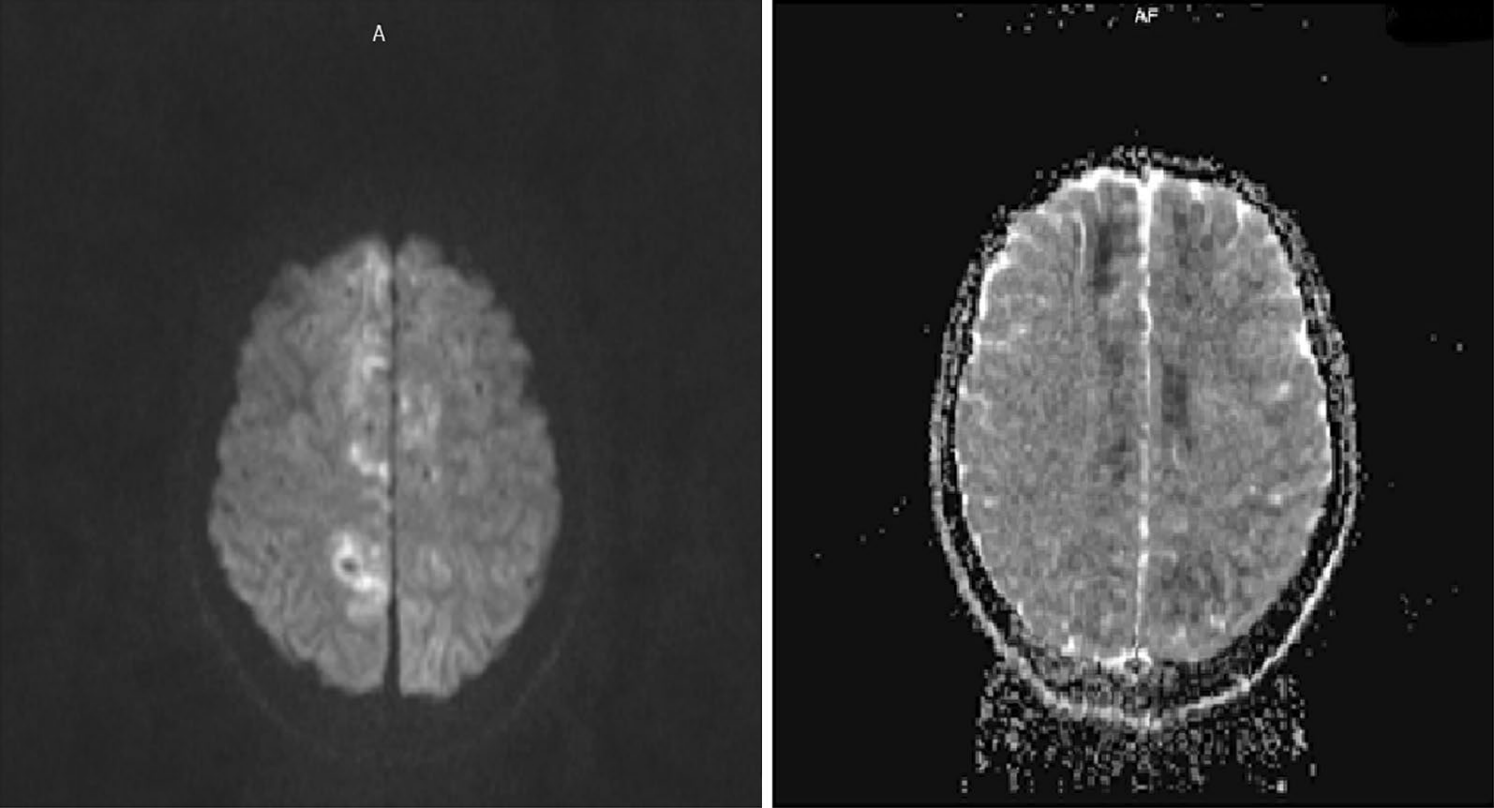
Axial diffusion weighted and ADC magnetic resonance imaging of the brain demonstrates bilateral cortical diffusion restriction in frontal, parietal and occipital lobes

Brain magnetic resonance imaging with venogram (Fig. 1) and angiogram (Fig. 2) MRI + MRV + MRA showed cerebral edema, bilateral and almost symmetric hyperintense T2 lesions with restricted diffusion in both frontal and occipital lobes (Fig. 3), and no underlying cerebral venous thrombosis CVT.

Lumbar puncture was performed and revealed the following results: 10 lymphocytes, elevated protein (50 mg/dl), no RBCs, and negative gram stain. Whereas blood glucose was 50 mg/dl, CSF glucose was 70.

Ordinary labs including Calcium, Albumin, MG, Na, K, creatinine, urea, LFTs, CBC, Plt, PT, PTT, and INR were all normal and troponin was negative.

The patient was admitted to the ICU and intubated.

Phenytoin at dose 15 mg/kg was loaded with close cardiac monitoring, and a maintenance dose of 100 mg twice daily was initiated in the second day.

A bolus (1 g/kg) of Mannitol was given intravenously, followed by a maintenance dose of 0.5 g/kg/8 h. GCS, consequently, improved to nine in 2 days.

The patient recovered completely and was discharged after a week of admission.

## Discussion

Lidocaine causes reversible blockade of impulse propagation along nerve fibers by preventing the inward movement (Fig. 2) of sodium ions through the nerve membrane. Local anesthetics of the amide type are thought to act at sodium channels of the nerve membrane. The addition of adrenaline (a vasopressor that works by stimulating both α- and β-adrenergic receptors) decreases the rate of absorption of lidocaine from the site of injection, increasing the duration of effect.

It has been proven that lidocaine has a dose dependent vascular effect. While low doses of lidocaine may cause vasoconstriction, moderate or high doses cause vasodilation and decreased SVR.

Local anesthetic medicines may have similar effects on excitable membranes in the brain and myocardium. However, central nervous system toxicity usually precedes the cardiovascular effects since it occurs at lower plasma concentrations.

The earliest signs of systemic toxicity are usually caused by blockade of inhibitory pathways in the cerebral cortex [8]. This allows for disinhibition of facilitator neurons resulting in excitatory cell overactivity and unopposed (generally enhanced) excitatory nerve activity, as a result, initial subjective symptoms of CNS toxicity include signs of excitation (Fig. 3) such as lightheadedness and dizziness, difficulty focusing, tinnitus, confusion, and circumoral numbness [9, 10]. Likewise, the objective signs of local anesthetic CNS toxicity are excitatory, for example, shivering, myoclonus, tremors, and sudden muscular contractions [11]. As the local anesthetic level rises, tonic–clonic convulsions occur. Symptoms of CNS excitations typically are followed by signs of CNS depression. Seizures activity ceases rapidly and ultimately is succeeded by respiratory depression and respiratory arrest.

Systemic toxicity could be precipitated not only by over dose or elevated injection rate, but also by accidentally injecting the local anesthetic into a highly vascularized area (intra arterial or intravenous accidental injection), which may have caused the toxicity in our patient as the gingival hematoma indicated.

The blood supply to the head and face comes partly via the external carotid artery, which branches off to form the internal maxillary artery. There are many proposals which advocate that accidentally injected local anesthetics into a branch of the external carotid artery may result in serious adverse effects by flowing in retrograde fashion down to the internal carotid artery and then directly to the brain.

Aldrete and Narang proposed that anesthetic solution injected accidentally into a branch of the external carotid artery could flow in a retrograde fashion so as to enter the internal carotid artery and gain direct access to the brain without having to pass first through the systemic circulation [12].

Aldrete and co-workers marshaled considerable evidence to support their “reverse carotid flow” theory. They found, for example, high concentrations of lidocaine in the internal carotid artery immediately after injecting the drug into the facial artery of dogs [13].

Several case reports have described such anesthetic toxicity in infants, experimental animals, and even in human adults after local anesthesia of brachial plexus, unintended intracarotid lidocaine injection during carotid endarterectomy surgery as well as after either epidural or caudal anesthetic injection. However, this is the first reported case of a status epilepticus developing after a dental procedure in a previously healthy patient.

Several differential diagnoses were plausible in this case. When in fact the paradox of CSF/serum glucose was explained by the effect of adrenaline, other CSF findings were compatible with either aseptic meningitis or encephalitis. Nonetheless, the absence of fever, the extremely acute presentation of seizures, the incompatibility of MRI findings, and the rapid spontaneous recovery made both of these differential diagnoses improbable. Thus, no further investigations like PCR were conducted.

Another important differential diagnosis was air embolism. However, there were many issues which argue against this diagnosis:

- The injected size of anesthetic solution was 1.5 ml, as a result, bubbles with a significant size were not bound to exist.
- If the solution had been injected intra arterially, repetitive seizures rather than a single seizure during the following half an hour would have been unlikely due to the little size and short lasted injection.
- If the solution had been injected intra venously, it would have been improbable that bubbles have overwhelmed the air-filtering capacity of the pulmonary vessels (>0.30 ml/kg/min) [14].

Though titration of serum level of lidocaine could have been beneficial, the small total injected dose −20 mg was too small to cause systemic toxicity in a previously healthy adult.

No follow up MRI was requested. However, a follow up brain CT scan was performed a week later and was completely normal. Consequently, MRI changes were probably transient changes due to the status epilepticus rather than stroke. Lidocaine induced vasoconstriction following distribution through systemic circulation was another possible mechanism of bilaterally restricted diffusion.

In this case, phenytoin was used safely to control seizures in spite of its potential for synergistic effect with lidocaine toward cardiac toxicity. Thus, phenytoin may be optimally avoided in such circumstances. Diazepam, however, represents the first line treatment while Phenobarbital comes second [15].

## Conclusion

It is vital for dentists to avoid injection of local anesthetics by aspirating before the injection and noting anatomical structures. They are also supposed to deeply understand the possible mechanisms of systemic toxicity. Dental professionals should recognize early signs of CNS toxicity of local anesthetics, including seizures and be prepared to manage them to optimize the patient outcome. Therefore, they should be well trained to do CPR, and it is recommended that suitable resuscitation equipment is available in dentistry clinics.

We have adhered to the CARE guidelines for completeness, transparency and data analysis in case reports.

## Abbreviations

CNS: central nervous system
ICU: intensive care unit
GCS: glasgow coma scale
ECG: electrocardiography
EEG: electroencephalography
CT: computed tomography
MRI: magnetic resonance imaging
MRV: magnetic resonance venography
CVT: cerebral venous thrombosis
CSF: cerebrospinal fluid
MG: magnesium
Na: sodium
K: potassium
LFTs: liver function tests
CBC: complete blood count
Plt: platelets
PT: prothrombin time
PTT: partial thromboplastin time
INR: international normalized ratio

## References

[1] Weinberg GL (2002). Current concept in resuscitation of patients with local anesthesic cardiac toxicity. Reg Anesth Pain Med.

[2] Long WB, Rosenblum S, Grady IP (1989). Successful rescuscitation of bupivacaine induced cardiac arrest using cardiopulmonary bypass. Amesth Analg..

[3] Moore DC, Bridenbaugh LD, Thompson GE, Balfour RI, Horton WG (1978). Bupivacaine: a review of 11,080 cases. Anesth Analg.

[4] Tomlin PJ (1974). Death in outpatient dental anaesthetic practice. Anaesthesia.

[5] DeToledo JC, Minagar A, Lowe MR (2002). Lidocaine-induced seizures in patients with history of epilepsy. Anesthesiology..

[6] Brown DL, Ransom DM, Hall JA, Leicht CH, Schroeder DR, Offord KP (1995). Regional anesthesia and local anesthetic-induced systemic toxicity. Anesth Analg..

[7] Menif K, Khaldi A, Bouziri A, Hamdi A, Belhadj S, Ben Jaballah N (2011). Lidocaine toxicity secondary to local anesthesia administered in the community for elective circumcision. Fetal Pediatr Pathol..

[8] Scott DB (1986). Toxic effects of local anesthetic agents on the central nervous system. Br J Anaesth..

[9] Scott BD (1975). Evaluation of the toxicity of local anesthetic agents in man. Br J Anesth..

[10] Mather LE, Tucker GT, Murphy TM, Stanton Hicks MD, Bonica JJ (1979). Cardiovascular and subjective central nervous system effects of long acting local anaesthetics in man. Anaesth Intensive Care.

[11] Scott DB (1979). Evaluation of clinical tolerance of local anaesthetic agents. Br J Anaesth.

[12] Aldrete JA, Narang R (1975). Deaths due to local analgesia in dentistry. Anaesthesia.

[13] Aldrete JA, Nicholson J, Sada T, Davidson W, Garrastasu G (1977). Cephalic kinetics of intra-arterially injected lidocaine. Oral Surg.

[14] Van Hulst RA, Klein J, Lachmann B (2003). Gas embolism: pathophysiology and treatment. Clin Physiol Funct Imaging.

[15] Drug toxcicity and metabolism in pediatrics. P88. 18, 19.

